# The Immunologic Role of Gut Microbiota in Patients with Chronic HBV Infection

**DOI:** 10.1155/2018/2361963

**Published:** 2018-07-25

**Authors:** Ruilin Yang, Yao Xu, Zhifeng Dai, Xuhong Lin, Huichao Wang

**Affiliations:** ^1^Department of Clinical Laboratory, Translational Medicine Center, Huaihe Hospital Affiliated to Henan University, Kaifeng, Henan 475000, China; ^2^Department of Nephrology, First Affiliated Hospital of Henan University, Kaifeng, Henan 475000, China

## Abstract

Hepatitis B can cause acute or chronic liver damage due to hepatitis B virus (HBV) infection. Cirrhosis or hepatocellular carcinoma (HCC) caused by chronic HBV infection often leads to increased mortality. However, the gut and liver have the same embryonic origin; therefore, a close relationship must exist in terms of anatomy and function, and the gut microbiota plays an important role in host metabolic and immune modulation. It is believed that structural changes in the gut microbiota, bacterial translocation, and the resulting immune injury may affect the occurrence and development of liver inflammation caused by chronic HBV infection based on the in-depth cognition of the concept of the “gut-liver axis” and the progress in intestinal microecology. This review aims to summarize and discuss the immunologic role of the gut microbiota in chronic HBV infection.

## 1. Introduction

Chronic hepatitis B virus (HBV) infection is a global problem that threatens human health. Chronic HBV infection can easily lead to some severe complications, such as liver failure, cirrhosis, and even hepatocellular carcinoma (HCC), which can be life-threatening if not treated in a timely and normative manner. According to the latest report from the World Health Organization (WHO), there are approximately 257 million people with chronic HBV infection worldwide, and of these cases, 887,000 people die due to complications caused by chronic HBV infection [1]. Although the application of the hepatitis B vaccine and antiviral drugs has caused the rate of HBV infection to decrease yearly, chronic HBV infection still remains a heavy economic burden and health threat for many families [2, 3].

Researchers have persisted in finding better ways to control this disease. In recent years, studies on intestinal flora have provided new targets for the prevention and treatment of chronic HBV infection. Studies have demonstrated that changes in the gut microbiota play important roles in inducing and promoting the development of liver diseases, and the diversity of the gut microbiota has been associated with alcoholic liver disease, autoimmune liver disease, chronic hepatitis B (CHB), cirrhosis and HCC; moreover, changes in the intestinal flora are dependent on the various pathogeneses that are present [4–8]. Furthermore, scholars from Taiwan have indicated that the gut microbiota may determine acute hepatitis B and CHB attacks. Specifically, the composition of the gut microbiota affects the host immune response to HBV, and the infection can easily transform into a chronic infection when the intestinal flora is in an abnormal state [9]. Accordingly, the fact that immune injuries caused by structural changes in the gut microbiota have a significant influence on the development of chronic HBV infection cannot be ignored.

## 2. Chronic HBV Infection and Innate Immunity

Adults generally experience acute onset after infection with HBV. However, most people are able to clear the virus via a healthy immune system, and only a few individuals with impaired immunity or other liver diseases progress to chronic HBV infection. Most chronic HBV infections occur in infants and young children due to their immature immune systems and unstable intestinal flora [10, 11]. Therefore, the occurrence and development of chronic HBV infection not only depend on the viral load, virulence, and invasion path of the HBV but also are associated with the immune function and intestinal flora of the host. It is currently believed that the cause of liver injury is not HBV replication in liver cells but rather the immune response caused by the HBV. The immune response is considered a key factor in the development of CHB. Various immune cells and cytokines not only are involved in the initiation and regulation of the immune response but also can activate downstream signaling pathways that directly or indirectly inhibit HBV replication. It can be concluded that the host immune response caused by HBV infection exerts a significant influence on the prognosis of hepatitis B and the treatment effect of antiviral drugs [12]. The immune response against HBV mainly includes innate immunity and adaptive immunity; however, the effective innate immune response not only can eliminate the virus directly but also can exert a significant influence on HBV-specific immunity [13].

Toll-like receptors (TLRs) are important protein molecules involved in innate immunity. Acting as a bridge connecting nonspecific immunity and specific immunity, they recognize molecules with conserved structures derived from microorganisms. After the breach of the body's physical barriers, such as the skin or mucous membranes by microorganisms, TLRs identify molecules and become activated, leading to immune responses [14]. TLRs are expressed in multiple liver immune cells, including plasmacytoid dendritic cells (pDCs), monocytes, macrophages, Kupffer cells, hepatic stellate cells, and liver cells [12]. Experimental studies have indicated that the activation of cell signaling pathways by TLRs and the release of antiviral cytokines are the main mechanisms by which HBV replication in liver cells is suppressed. When the host is infected by HBV, the virus is identified by the relevant TLRs, and the mechanisms of antiviral regulation are activated. These mechanisms include TLR recognition, the release of interferon (IFN), activation of NK and NKT cells, and the production of proinflammatory cytokines [14]. For example, the activation of the HBV-TLR3 signaling pathway causes the production of IFN-*α*/*β*, and TLR2/TLR4 can initiate the MAPK and PI3K/Akt signaling pathways [15]. However, another research has demonstrated that HBV can reduce the expression of TLR2 in liver Kupffer cells and mononuclear cells in patients with chronic HBV infection and can also attenuate intracellular signaling pathways to enable the evasion of the immune response [16]. A recent study also found that the HBV DNA viral load is negatively correlated with TLR7 expression in biopsy samples, which indicates that expression of TLR7 on liver cells can be inhibited by HBV [17]. Obviously, the TLR-mediated signaling pathways induce immune responses to HBV infection, but the HBV virus itself downregulates the expression of TLRs on the immune cells. The latter mechanism may be one of the main causes of progression to chronic HBV infection in patients. Studies on the gut-liver axis have revealed that the intestine and liver have the same embryonic origin and are linked by the portal venous system. The intestinal tract has been proposed to exert a regulatory effect on the development of chronic HBV infection. The intestinal flora is an important mediator of the interaction between the intestine and the liver and the portal vein functions as a bridge connecting these structures [8].

## 3. Gut Microbiota

As the name suggests, the gut microbiota includes microorganisms that have colonized and coexist in the human intestinal tract. Due to host genetic, diet, and environmental factors and use of antibiotics, certain differences exist in the components of the human intestine among individuals, but the human body will gradually establish a stable intestinal flora structure and regulate and maintain the body's health under the influence of these factors [18–20]. Tens of thousands of microbes live in the intestinal tracts of normal adults, and most of these microbes are located in the colon. The number of bacteria located in the colon of the human body is estimated to be approximately 3.8 × 10^13^ according to the identified bacterial content and colon volume. There are approximately 40,000 types of microbes in the gut and can be divided into two main categories, that is, *Bacteroides* and the thick-walled bacteria. The most common bacteria are *Bifidobacteria*, *Lactobacillus*, *Clostridium*, and *Streptococcus* [21]. These intestinal bacteria are considered beneficial, and their roles in metabolism, immunity, and nutrition can prevent invasion of the host by disease-causing pathogens [22]. Thus, the intestinal immune function cannot be neglected. Data of experiments showed that imbalances of the intestinal flora easily lead to disorders of the intestinal immune system and cause a variety of diseases such as irritable bowel syndrome (IBS), inflammatory bowel disease (IBD), autoimmune liver disease, and CHB [23–26]. Moreover, the gut microbiota is involved in nonalcoholic fatty liver disease (NAFLD) and associated with the progression of NAFLD to nonalcoholic steatohepatitis, cirrhosis, or HCC [19, 27].

## 4. Immune Function of Gut Microbiota in the Development of Chronic HBV Infection

In recent years, many scholars, especially domestic scholars, have studied the relationship between gut microbiota and chronic HBV infection because of the high proportion of HBV-infected people worldwide. In 2006, Xing et al. found that liver ischemia and reperfusion can reduce the numbers of intestinal *Bifidobacteria* and *Lactobacillus* and increase the numbers of Enterobacteriaceae and *Enterococcus*; these changes are associated with the loss of intestinal microvilli, the widening of the intestinal mucosal space and intestinal bacterial translocation [28]. Subsequently, the same changes were found in the intestinal flora of chronic HBV carriers, CHB patients, and hepatitis B-induced cirrhosis patients; that is, the structures and abundances of the bacterial groups were obviously different. Specifically, patients with CHB and cirrhosis exhibit dramatically decreased *Bifidobacteria* and *Lactobacillus* levels, while *Enterococcus* and Enterobacteriaceae levels are significantly increased compared to healthy people. The progression of liver diseases, particularly liver cirrhosis, is caused by bacterial products from the intestine [29, 30]. Chou et al. found that HBV could not be detected after six weeks of HBV infection in adult mice without intestinal flora (treated by antibiotics), and 60% of adult mice with intestinal flora (no antibiotics) still exhibited HBV. These data imply that gut microbes play a critical role in immunity against HBV [9]. Wang et al. studied the intestinal flora of CHB patients and healthy people, and the data revealed that the *Bacteroides* level was decreased in CHB patients compared to healthy people based on sequencing the V3-V4 region of the 16S rRNA gene of the intestinal flora. Additionally, these authors also discovered that the intestinal microflora structure of the CHB patients had changed compared to that before severe liver injury, which indicated that the structural changes in the intestinal flora played a potential pathogenic role in patients with chronic HBV infection [31]. In summary, an imbalance exists in the intestinal microbiota of patients with CHB and cirrhosis. Similarly, overgrowth of harmful bacteria in the intestinal tract leads to increased mucosal permeability, which causes harmful bacteria to travel through the portal vein into the liver and thus activate the liver's innate immune system. The structural changes in the intestinal microflora and the severity of liver disease are mutually causal, and to a certain extent, they affect the transformation process of CHB to liver fibrosis or liver failure [12]. Therefore, we suggest that, in chronic HBV infection, the injury to hepatocytes not only originates from the cellular immune response caused by HBV invasion but also is caused by the natural immune response elicited by pathogen-associated molecular patterns (PAMPs) produced by the intestinal microbes with structural disorders. TLRs are the main pattern recognition receptors in the natural immune system and play a crucial role in the immune response [12]. It is now known that the intestinal PAMPs associated with chronic HBV infection are mainly composed of lipopolysaccharide (LPS), unmethylated CpG DNA, bacterial cell wall components, and bacterial DNA/RNA. The related immune mechanisms are described below.

### 4.1. The LPS-TLR4 Pathway

LPS is the main component of the outer membrane of Gram-negative bacteria and is an endotoxin mainly released by Enterobacteriaceae. Research has demonstrated a high level of LPS in the blood of patients with chronic HBV liver failure, which indicates that LPS may be related to the severity of the disease [32]. Another study found that LPS in the intestinal tract can downregulate the expression of tight junction proteins (ZO-1 and closed protein), increase the permeability of the intestinal mucosa, and enter the blood flow through the portal venous system [33, 34]. LPS can be recognized by TLR4, and TLR4 is mainly expressed in mononuclear macrophages [32]. Chou et al. [9] found that mice, regardless of age, subjected to TLR4 silencing are able to clear HBV and obviously produce antibodies in 8 weeks. In other words, TLR4-silenced mice are not immune to LPS but can clear HBV. Conversely, LPS-induced immune responses may contribute to the progression of chronic HBV infection. Researches showed that LPS binds to LPS-binding protein, and this combination can be identified by TLR4 on the surface of mononuclear macrophages. This recognition then stimulates CD14^+^ Kupffer cells, triggers the inflammatory cascade effect, activates the NF-*κ*B-related pathway, and produces inflammatory cytokines, such as tumor necrosis factor-*α* (TNF-*α*), IL-1, and IL-6, and thus causes acute liver injury [35, 36]. Simultaneously, this pathway also induces Kupffer cells to release immunosuppressive mediators, such as IL-10, which can inhibit the release of inflammatory mediators of mononuclear macrophages and HBV-specific immune responses, in turn inhibiting the efficient clearing of bacteria and HBV [37]. Additionally, hepatic stellate cells also express TLR4 and can release a large number of extracellular matrix proteins in a LPS-TLR4 pathway-dependent manner. These proteins are involved in the fibrotic process and may also be among the factors that cause chronic HBV infection to develop into liver fibrosis [38].

### 4.2. The Unmethylated CpG DNA-TLR9 Pathway

Unmethylated CpG DNA is an important immune adjuvant that can activate TLR9. TLR9 is mainly expressed on mononuclear cells, B cells, CD4^+^ T cells, pDCs, and Treg cells [39]. The CpG-TLR9 signaling pathway not only activates the innate immune response but also adjusts the adaptive immune response. The CpG-TLR9 signaling pathway plays an important role in the prevention and treatment of infectious diseases [40]. Researches have highlighted that CpG-TLR9 and MyD88 form compounds (MyD88, IRAK4, and TRAF6), and TRAF6 phosphorylates IRAK1 to trigger the NF-*κ*B and MAPK signaling pathways, which may activate DCs to express and secrete cytokines and chemokines to further promote the proliferation and differentiation of B and T cells, resulting in the secretion of proinflammatory cytokines and IFN [40, 41]. However, different bacteria have different CpG DNA levels. In the intestinal flora of animals, unmethylated CpG DNAs are abundant in *Lactobacillus casei*, *Lactobacillus plantarum*, *Lactobacillus rhamnosus*, *Bifidobacteria*, Proteobacteria, and Bacteroidetes [40]. As mentioned above, the intestinal floras of chronic HBV-infected patients with aggravated conditions are highly maladjusted, and *Lactobacillus* and *Bifidobacteria* with richer unmethylated CpG DNA levels are greatly reduced, causing the weakening of the CpG DNA-TLR9 pathway, decreasing of the production of protective cytokines, especially IFN, and diminishing of the immune effect on HBV.

### 4.3. Other Pathways

Other components of intestinal bacteria can also be identified by liver immune cells, such as the following cell wall components: teichoic acid (TA), peptidoglycan (PGN), and specialized proteins (flagellin). TA and PGN are recognized by TLR2, and flagellin mainly activates TLR5 [42, 43]. TLR3 can combine with dsRNA in bacteria, and ssRNA can activate TLR7 and TLR8 receptors [44]. A series of protective immune responses depend on the MyD88-TRIF pathway, which triggers the downstream signal and then initiates the activation of related immune cells to release proinflammatory cytokines [45]. Nevertheless, this process will also aggravate liver injury if the immune response persists for a long time or reaches an excessive intensity, which then results in the production of a large number of cytokines in the body [32].

## 5. Summary and Prospects

In conclusion, studies have confirmed that imbalances of the intestinal flora play an important role in promoting the development of chronic HBV infection. The PAMPs of intestinal bacteria are transferred into the liver through the portal vein and identified by TLRs in the immune cells, which causes a series of immune responses, the release of various cytokines (IL, TNF, and IFN), and further liver cell damage. Among these processes, TLRs play an essential role as an important link between the intestinal flora and the liver immune reaction.

Although antiviral drugs and IFN have significant efficacy in the treatment of chronic HBV infection, for various reasons, it seems inevitable that chronic HBV infection develops into liver cirrhosis, liver failure, or liver cancer. Currently, studies on the gut microbiota and its products have provided a new therapeutic target. An article proposed that the application of synthetic unmethylated CpG DNA is a promising measure for the treatment of infectious diseases [41]. Additionally, the physiological indexes (BMI) and serum metabolites of the patients with chronic HBV infection also correlated with the structure of the intestinal flora and liver fibrosis [31]. Certain physical exercises may actively regulate the intestinal floras of patients with chronic diseases [46].

In recent years, probiotics have been widely used in the treatment of intestinal diseases. Many studies have demonstrated that compound probiotics can improve the abnormal state of the gut microbiota, as well as chronic inflammation in chronic liver diseases, but short-term probiotic treatment still has no effect on adjusting intestinal permeability or liver function [47, 48]. With the in-depth study of gut microbiota, someone suggested that fecal microbiota transplantation (FMT) will be a promising treatment for chronic HBV infection. FMT refers to the infusion of a faecal suspension from healthy people into the intestinal tract of patients to cure a specific disease [32]. More and more researches showed that FMT has been used to treat *Clostridium difficile* infection, IBD, IBS, and various liver diseases with some effects [49]. A 2015 case report described that FMT can reduce blood ammonia, increase cognitive abilities, and improve hepatic encephalopathy for a 57-year-old male with cirrhosis secondary to both alcohol and the hepatitis C virus [50]. A report on HBeAg-positive CHB therapy in patients with ongoing ETV/TDF therapy showed that FMT can induce HBeAg clearance in a significant proportion of cases who have persistent positive HBeAg even after long-term antiviral treatment. Although only 5 patients participated in the FMT group, the statistics varied significantly (*P* = 0.0001) and these dramatic results were particularly encouraging for patients with HBeAg-positive CHB who could not cease oral antiviral therapy [51]. Therefore, FMT may be as a potential immunomodulator in improving the intestinal microenvironment and alleviating the damage to the liver caused by harmful intestinal bacteria [32]. However, there are also associated ethical, legal, and social problems; we should establish a balance of scientific research, health, and marketing regarding the study of the intestinal flora [52]. And the data on the field of FMT treatment related to HBV-related diseases is still limited. So there will be a lot of work to do on FMT and chronic HBV infection for us in the future [32].
